# Efficacy Analysis of Combinatorial siRNAs against HIV Derived from One Double Hairpin RNA Precursor

**DOI:** 10.3389/fmicb.2017.01651

**Published:** 2017-08-29

**Authors:** Chang Liu, Zhipin Liang, Xiaohong Kong

**Affiliations:** Medical Molecular Virology Laboratory, School of Medicine, Nankai University Tianjin, China

**Keywords:** HIV, RNAi, gene therapy, long-term inhibition, escaping mutation

## Abstract

Combinatorial small interfering RNA duplexes (siRNAs) have the potential to be a gene therapy against HIV-1, and some studies have reported that transient combinatorial siRNA expression represses HIV replication, but the effects of long-term siRNA expression on HIV replication have not been studied in detail. In this study, HIV-1 replication under the influence of stable combinatorial siRNA expression from a single RNA transcript was analyzed. First, a series of cassettes encoding short hairpin RNA (shRNA)/long hairpin RNA (lhRNA)/double long hairpins (dlhRNA) was constructed and subjected to an analysis of inhibitory efficacy. Next, an optimized dlhRNA encoding cassette was selected and inserted into lentiviral delivery vector FG12. Transient dlhRNA expression reduced replication of HIV-1 in TZM-bl cells and CD4+ T cells successfully. HIV-1 susceptible TZM-bl cells were transducted with the dlhRNA expressing lentiviral vector and sorted by fluorescence-activated cell sorting to obtain stable dlhRNA expressing cells. The generation of four anti-HIV siRNAs in these dlhRNA expressing cells was verified by stem–loop RT-PCR assay. dlhRNA expression did not activate a non-specific interferon response. The dlhRNA expressing cells were also challenged with HIV-1 NL4-3, which revealed that stable expression of combinatorial siRNAs repressed HIV-1 replication for 8 days, after which HIV-1 overcame the inhibitory effect of siRNA expression by expressing mutant versions of RNAi targets. The results of this evaluation of the long-term inhibitory effects of combinatorial siRNAs against HIV-1 provide a reference for researchers who utilize combinatorial RNA interference against HIV-1 or other error-prone viruses.

## Introduction

Acquired immunodeficiency syndrome (AIDS) is a global epidemic disease that is caused by the human immunodeficiency virus (HIV) and results in destruction of the immune system and death as a result of opportunistic infection. Highly active antiretroviral therapy (HAART) is the most commonly used and effective therapy against HIV-1 (Woods and Foisy, 2014; Margolis et al., 2016). Although HAART can effectively control HIV-1 infection and slow the progression of the disease, it usually requires a life-long commitment and it is associated with some comorbidities (Pennings, 2013; Passaes and Saez-Cirion, 2014). Thus, effective alternative therapies against HIV, such as RNA interference (RNAi)-based gene therapies, hold significant promise with regard to lengthening and improving the lives of patients infected with HIV-1 (McIntyre et al., 2009; Saayman et al., 2010; Philippidis, 2013; Spanevello et al., 2016).

RNA interference (RNAi) is an evolutionarily conserved gene silencing mechanism in eukaryotes. RNAi plays an important role in regulating gene expression and innate antiviral immune responses (Meister and Tuschl, 2004; Haasnoot et al., 2007), and it is widely used to suppress cellular or viral genes at the post-transcriptional level (Mollaie et al., 2013; Deng et al., 2014). Double-stranded RNAs serve as precursors from which small interfering RNAs (siRNAs) are derived. However, in laboratory applications, short hairpin RNAs (shRNAs) are generally used to induce RNAi in mammalian cells (Brummelkamp et al., 2002; Paddison et al., 2002). Like double-stranded RNAs, shRNAs are processed by Dicer endonuclease into siRNA duplexes, which are loaded into the RNA-induced silencing complex (RISC). Following degradation of the passenger strand of the siRNA duplex, the guide (targeting) strand binds to a complementary sequence on a target mRNA, which is subsequently degraded by RISC, thus preventing translation (Meister and Tuschl, 2004).

Due to the high mutation rate of HIV-1, guide RNAs usually correspond to highly conserved regions of the viral genome (Naito et al., 2007; von Eije et al., 2008), and several siRNAs are always targeted against different mRNAs simultaneously (Liu et al., 2007, 2009; Saayman et al., 2010). Several types of combinatorial siRNAs have been reported, including extended short hairpin RNAs (e-shRNAs), long hairpin RNAs (lhRNAs), double long hairpin RNAs (dlhRNAs), and other long double-stranded RNA expression cassettes. All of these RNA precursors have a special RNA secondary structure that allows them to be processed into several different siRNAs by the intracellular RNAi machinery. In contrast to methods involving expression of several shRNAs separately under different promoters, which usually involve competing with microRNAs and disrupting normal cellular RNAi pathways, combinatorial siRNA methods can generate different siRNAs from a single RNA precursor and do not induce a non-specific interferon response because of the specific secondary structure of these hairpin RNAs. Several studies have demonstrated that transient expression of appropriate long double-stranded RNAs inhibits HIV replication (Liu et al., 2009; Saayman et al., 2010), but the long-term inhibitory effect of stable siRNA expression has not been studied comprehensively. Furthermore, some studies have reported that long-term combinatorial RNAi produced only transient inhibition of HIV-1 replication (Westerhout et al., 2005; Shah et al., 2012).

In this study, we investigated the effect of long-term expression of dlhRNA-derived combinatorial siRNAs against four different HIV-1 genes (*gag, tat, vpu*, and *env*) on HIV-1 replication. We performed the following steps: (i) selecting four conserved RNAi targets against HIV-1, followed by constructing and evaluating the efficacy of four corresponding shRNA expression cassettes; (ii) constructing and selecting optimized lhRNA and dlhRNA expression cassettes successively, followed by analyzing the RNAi efficacy of selected dlhRNA expression cassettes; (iii) constructing a lentiviral vector to deliver dlhRNA and testing its inhibitory effect on HIV-1 replication; (iv) sorting HIV-susceptible cells that stably expressed the dlhRNA and analyzing the long-term inhibitory effect of the dlhRNA on HIV replication in these cells.

## Materials and Methods

### Cells, Plasmids, and Viruses

TZM-bl and HEK293T cells were cultured in Dulbecco’s modified Eagle’s Medium (DMEM; Invitrogen, Paisley, United Kingdom), supplemented with 10% fetal bovine serum (FBS; HyClone, Logan, UT, United States), 100 U/mL penicillin (HyClone), and 100 μg/mL streptomycin (HyClone) at 37°C with 5% CO_2_.

PBMCs were obtained from healthy adult volunteers under an IRB approved protocol. CD4+ cells were purified from PBMCs using CD4+ T cell enrichment kits (StemCell Technologies). CD4+ T cells were cultured in RPMI 1640 medium supplemented with 10% fetal bovine serum (FBS; HyClone, Logan, UT, United States), 100 U/mL penicillin (HyClone), and 100 μg/mL streptomycin (HyClone) at 37°C with 5% CO_2_.

HIV/eGFP fusion protein expression plasmids were constructed to measure the efficacy of RNA interference. The primers used to amplify the *gag, tat, vpu*, and *env* genes are shown in Supplementary Table 1. The *gag/tat/vpu/env* gene segment was inserted into the peGFP-N1 plasmid to construct HIV/eGFP fusion gene expression plasmids pGag-eGFP, pTat-eGFP, pVpu-eGFP, and pEnv-eGFP.

The RNAi plasmids with shRNA/lhRNA/dlhRNA expression cassettes were designated pGag-shRNA, pTat-shRNA, pVpu-shRNA, pEnv-shRNA, pGag-Tat-lhRNA, pTat-Gag-lhRNA, pGag-Vpu-lhRNA, pVpu-Gag-lhRNA, pEnv-Tat-lhRNA, pTat-Env-lhRNA, pEnv-Vpu-lhRNA, pVpu-Env-lhRNA, pTEVG-dlhRNA, and pVGTE-dlhRNA. The construction method was performed according to the two-step PCR method described by Castanotto et al. (2002) and Saayman et al. (2010). RNAi-Ready pSIREN-RetroQ (Clontech, No. 631526) was used as a template to obtain the human U6 promoter. The primers used for shRNA/lhRNA/dlhRNA construction are listed in Supplementary Table 2.

Lentiviral vector packaging system helper plasmid pMDLg/pRRE contained the *gag* and *pol* genes of HIV-1; however, the *gag* gene was also a target in our research. To prevent self-repression of *gag*, synonymous mutation plasmid pMDLg/pRRE-M was constructed. Using a site-directed mutagenesis kit (Finnzymes, F-541), the sequence “gaaggagccaccccacaagattt” was mutated into “gaGggCgcTacAccTcaGgaCttt” (mutation sites are marked in capital letters). All of the constructed plasmids were verified by sequencing. Lentiviral expression vector plasmid FG12-VGTE was sub-cloned from pVGTE-dlhRNA.

For HIV virion harvesting, HIV-1 infectious clone plasmid pNL4-3 (GenBank: AF324493.2) was transfected into HEK293T cells using polyethylenimine (PEI) (Polyscience, Cat:23966). The viral supernatant was harvested at 60 h post-transfection, filtered through a 0.45-μm filter, dispensed, and stored at -80°C. The 50% tissue culture infective dose (TCID50) of the virus stock was determined by infecting TZM-bl cells with fourfold serial dilutions of virus (Kong et al., 2008).

### RNA Interference Assay

An HIV/eGFP fusion protein reporter system was used to test the inhibitory efficacy of the shRNA/lhRNA/dlhRNA expression cassettes (Liang et al., 2012). A decrease in the GFP positive ratio between the RNA expression cassette and scramble-RNA group was taken as evidence of inhibition. The RNAi plasmid (1 μg) and HIV/eGFP fusion gene expression plasmid (1 μg) were co-transfected into HEK293T cells (5 × 10^5^ cells in each well of a 6-well cluster). After 48 h, cells were observed under a microscope, images were collected, and cells were harvested for flow cytometry. Mock-transfected cells were used as a negative control. The DMEM was discarded, after which the cells were digested for 3 min with 500 μL per well of 0.25% trypsin. The reaction was terminated with 500 μL DMEM (10% serum) and the mixture was mixed by pipette. The cell suspensions were pooled in a 1.5 mL tube, after which the cells were pelleted at 850 × *g* for 5 min. The supernatant was discarded, after which the pellet was suspended in 500 μL phosphate buffer solution (PBS). Cells were pelleted again at 850 × *g* for 5 min, the supernatant was discarded, and the pellet was fixed in 500 μL 1% paraformaldehyde for 30 min in the dark. Finally, the proportion of green fluorescent cells (GFPs) was checked using a flow cytometer (FACSCalibur^TM^, BD).

### Lentiviral Particle Packaging

HEK293T cells (1 × 10^7^ cells in a 15-cm dish) were co-transfected with lentiviral vector plasmid pFG12 or pFG12-VGTE (25 μg) and helper plasmids (6.25 μg pRSV-rev, 12.5 μg pHCMV-G, and 7.5 μg pMDLg/pRRE-M). The plasmids and PEI (Polyscience, 23966) were mixed for 10 min in serum-free DMEM at a ratio of 1:4. The medium was refreshed at 8 h post-transfection. The culture supernatant was harvested 48 h after transfection, filtered through a 0.45-μm filter, and concentrated via ultracentrifugation (Beckman, LE-80K) at 82,000 × *g* for 1.5 h at 4°C. The supernatant was discarded gently after ultracentrifugation, and 200 μL serum-free DMEM was added to the tube. The pellet was suspended overnight at 4°C. On the next day, the medium containing the lentiviral particles was dispensed into a 25-μL per tube and stored at -80°C for further study. The lentiviral particle titer was determined by infecting TZM-bl cells with serial dilutions of the stock solution. The lentiviral particle titer was generally approximately 10^8^ transducing units (TU)/mL.

### Quantitative Real-time RT-PCR

To evaluate the siRNA cleavage efficiency of the dlhRNA-encoding cassette, 2 μg of the dlhRNA-encoding plasmid or the corresponding shRNA-encoding plasmid was transfected into HEK293T cells (5 × 10^5^ per well in a 6-well culture plate). Total RNA was extracted from the HEK293T cells at 48 h post-transfection using TRIzol Reagent (Life Technologies, 15596-026) according to the manufacturer’s instructions. RNA pellets were suspended in 30 μL nuclease-free water, after which the RNA concentration was determined. RT- PCR was performed according to Chen’s stem–loop RT-PCR method (Chen et al., 2005). All RT primers are listed in Supplementary Table 3. After cDNA of U6a, Gag-RNAi, Tat-RNAi, Vpu-RNAi, and Env-RNAi was obtained, 1 μL of cDNA was analyzed by qPCR in triplicate using SuperReal PreMix Plus (SYBR Green) (Tiangen, FP204-01). The primers used in these experiments are listed in Supplementary Table 3. Real-time PCR was performed as follows: denaturing for 15 min at 95°C, followed by 40 cycles of 95°C for 10 s, 56°C for 30 s, and 72°C for 30 s. The specificity of the PCR amplification was also verified by melting curve analysis.

To assess activation of the IFN response in cells expressing dlhRNA, interferon beta (IFN-β) mRNA was measured using a sensitive quantitative PCR assay. Total RNA was extracted using TRIzol Reagent (Life Technologies, 15596-026) according to the manufacturer’s instructions from cells expressing dlhRNA. Double-stranded RNA Poly I:C (50 μg/mL, Sigma, St. Louis, MO, United States)-treated cells were used as a positive control group, and non-treated TZM-bl cells were used as a negative control group. PCR was performed with an oligo-dT (18T) primer as described above (Takara, D2639A). After reverse transcription, 1 μL of cDNA was analyzed by qPCR in triplicate using SuperReal PreMix Plus (SYBR Green) (Tiangen, FP204-01). The primers used for qPCR are listed in Supplementary Table 3. The PCR conditions were as follows: 95°C for 15 min followed by 40 cycles of 95°C for 10 s, 60°C for 30 s, and 72°C for 30 s. GAPDH mRNA was used as a loading control. The specificity of the PCR products was also verified by melting curve analysis.

### Fluorescence-Activated Cell Sorting (FACS)

To evaluate the long-term inhibitory effect of the VGTE-dlhRNA expression cassette against HIV, HIV-1-susceptible TZM-bl cells were transducted by lentiviral particles FG12-VGTE or FG12. Approximately, 5 × 10^5^ TZM-bl cells were infected by FG12-VGTE or FG12 with a multiplicity of infection (M.O.I.) of 10 for 72 h in a 10-cm dish. Three days post-transduction, cells were sorted via live FACS (BD, FACSAria II), and cells expressing GFP were selected. The selected cells were designated as FG12-VGTE-TZM-bl or FG12-TZM-bl cells. Cells collected by FACS were incubated for 48 h before infection with HIV-1 NL4-3.

### HIV Replication Assay

Replication of HIV-1 NL4-3 was evaluated in TZM-bl cells by luciferase assay as previously described (Liang et al., 2012). Briefly, TZM-bl cells were seeded in 96-well plates (4 × 10^3^ cells in each well) and infected with HIV-1 NL4-3 (M.O.I. = 0.01) for 2 h. Cells in the negative control (NC) group were mock-infected. Cells were washed three times with 100 μL PBS per well. FG12-VGTE and FG12 lentiviral particles were added separately with varying M.O.I. (0.01, 0.02, or 0.04). NC group cells and HIV group cells were not treated with lentiviral particles. Additional FG12-VGTE or FG12 lentiviral particles were added every 24 h for a total of four exposures. Cells were harvested for measurement of luciferase activity 16 h after the last treatment. The luciferase assay was performed according to the rapid protocol for the Steady-Glo assay system (Promega, E2520) with a GloMax^®^96 Microplate Luminometer (Promega) according to the manufacturer’s instructions. Relative luciferase activity (RLA) was used to reflect HIV-1 replication.

Replication of HIV-1 NL4-3 in CD4+ cells was analyzed by p24 ELISA assay (Perkin Elmer) according to the manufacturer’s instructions. Briefly, 2 × 10^5^ CD4+ cells were activated with PHA (2 μg/mL) before transduction with corresponding lentiviruses (FG12-VGTE or FG12) at M.O.I. of 20. 48 h after activation, cells were infected with NL4-3 at M.O.I. of 0.01. Supernatants from CD4+ T cells were collected and analyzed for HIV replication by p24 ELISA assay.

For the HIV-1 replication kinetics assays using FG12-VGTE-TZM-bl cells or FG12-TZM-bl cells, 2 × 10^4^ cells per well were seeded in a 24-well plate. All cells were infected by HIV-1 with an equivalent M.O.I. of 0.01. After incubation at 37°C overnight, cells were washed three times with PBS, after which 800 μL of fresh DMEM was added. A 400-μL aliquot of each infected culture was sampled on days 0, 2, 4, 6, 8, 10, 12, and 14. The culture medium was replenished with an equal volume of fresh DMEM after each sample was collected. To determine the viral yield at each time point, 50 μL of each culture supernatant was used to infect 2 × 10^4^ TZM-bl cells in a 96-well plate. At 48 h post-infection, luciferase activity and titration assays were performed as described above.

### Statistical Assay

The statistical significance between two different groups of each inhibition efficiency assay was determined by the Student paired *t-*test. *P*-values of ≤ 0.05 were considered to be significant.

## Results

### Construction and Functional Assays of shRNA/lhRNA/dlhRNA Expression Cassettes

**FIGURE 1:**
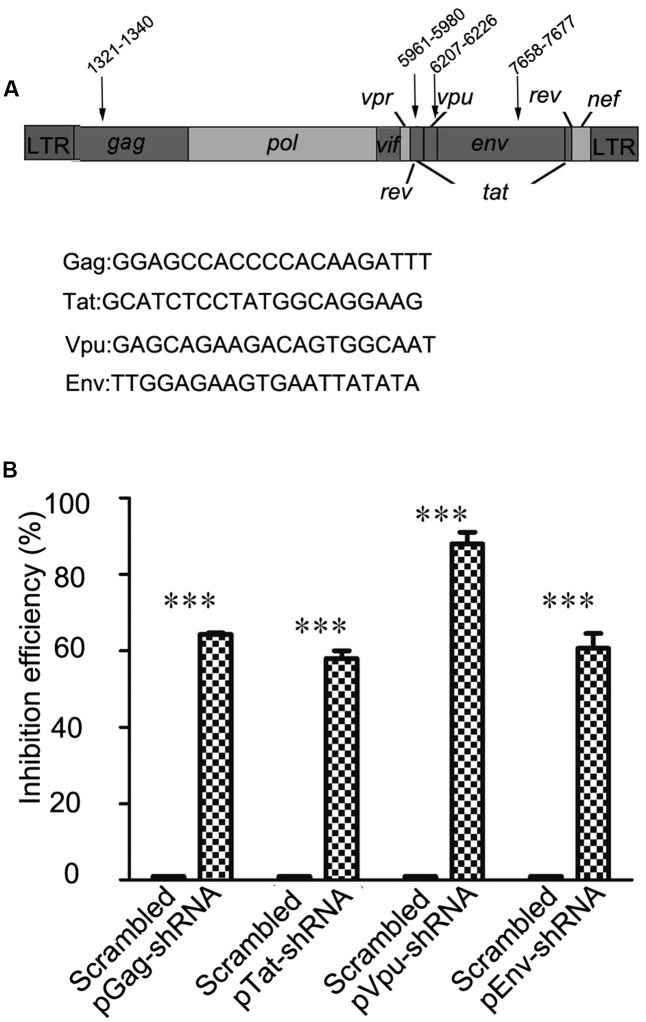
Selection of four RNAi targets and functional assessment against HIV-1. **(A)** The location and sequence information of four RNAi targets; the numbering positions in HIV are relative to HXB2CG. Four HIV-1 genes were selected as targets: *gag, tat, vpu*, and *env*. **(B)** The relative reduction in the expression level of the HIV gene/eGFP fusion protein was taken as the inhibitory efficacy of RNAi. In comparison with scrambled shRNA, the Gag, Tat, Vpu, and Env shRNAs repressed expression of the corresponding HIV gene/eGFP fusion proteins by 64.3, 58, 88, and 60.7%, respectively. ^∗∗∗^*p* < 0.001, compared with the scrambled shRNA-treated control group. All experiments were performed more than three times.

In order to choose RNAi targets with the potential to alter HIV-1 replication, we consulted previous studies and online HIV-1 resources, including HIV Databases^1^ and the siVirus online tool^2^ (McIntyre et al., 2009). Finally, four highly conserved RNAi targets located in two structural genes (*gag* and *env*) and two non-structural genes (*tat* and *vpu*) were chosen as shown in **Figure 1A**. Long terminal repeat (LTR) sequences, most of which are highly conserved, were not chosen for our study, because RNAi targets have been identified in the LTR sequence of the FG12 lentiviral vector applied in this study. Four shRNA expression cassettes were designed and constructed according to the target gene sequences. As shown in **Figure 1B**, flow cytometry assay results showed that each of the four shRNA expression cassettes decreased expression of its corresponding viral gene-eGFP fusion protein (64.3% for Gag-shRNA, 58% for Tat-shRNA, 88% for Vpu-shRNA, and 60.7% for Env-shRNA).

**FIGURE 2:**
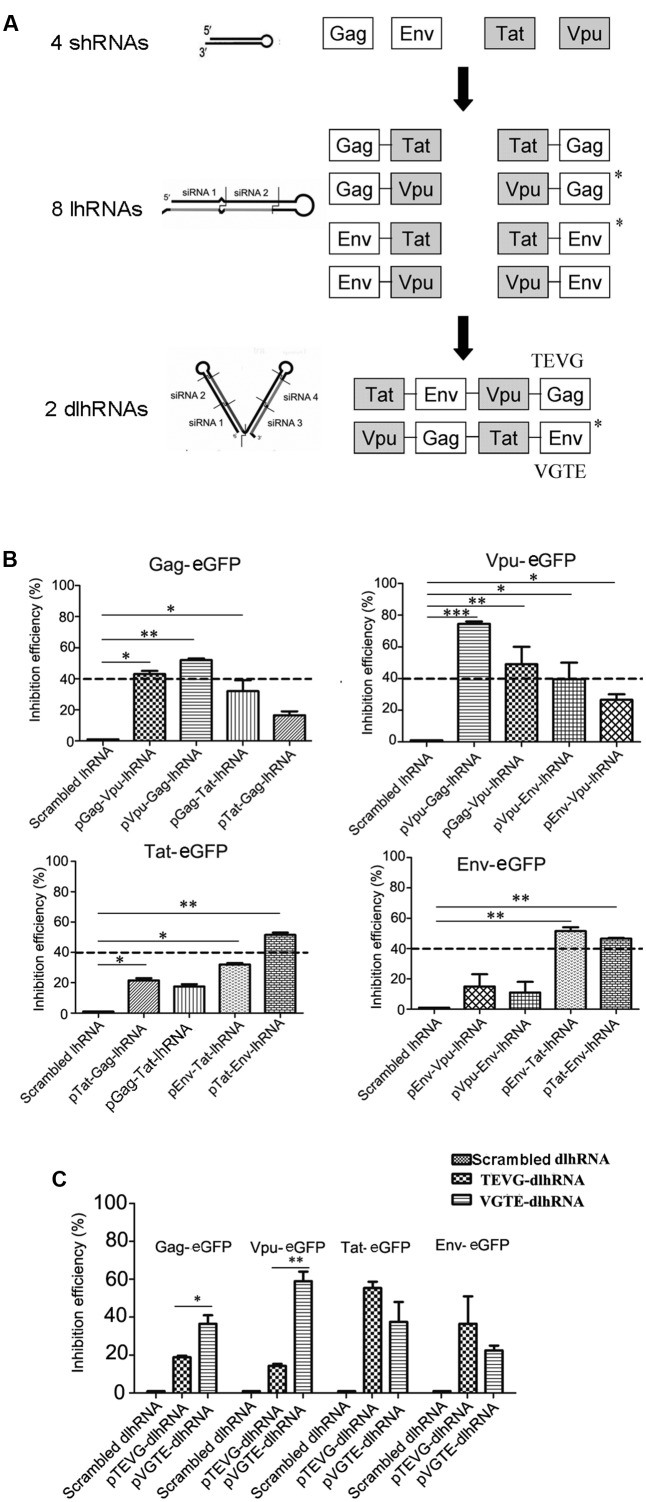
Selection and functional assessment of the optimized dlhRNA expression cassette. **(A)** Flowchart of shRNA/lhRNA/dlhRNA expression cassette construction and selection. White boxes show effective segments against HIV-1 structural genes (*gag* and *env*); gray boxes show effective segments against non-structural genes (*tat* and *vpu*). Asterisks indicate selected lhRNA/dlhRNA-encoding cassettes. **(B)** Inhibition efficacy assay of the lhRNA expression cassettes. Dashed lines indicate the selection standard (40% inhibition efficiency). ^∗^*p* < 0.05, ^∗∗^*p* < 0.01, ^∗∗∗^*p* < 0.001, difference compared with the scrambled shRNA treated control. **(C)** Inhibition efficacy assay of the dlhRNA expression cassettes. The assay was performed similarly to the inhibition efficacy assays of shRNA and lhRNA. ^∗^*p* < 0.05, ^∗∗^*p* < 0.01, VGTE group vs. TEVG group. All experiments were performed more than three times.

Based on these results, a series of lhRNA and dlhRNA expression cassettes were constructed and assessed with regard to RNAi efficacy following the process shown in **Figure 2A**. Eight lhRNA-encoding cassettes, each containing a structural gene RNAi target and non-structural gene RNAi target, were selected from the combinations of structural gene RNAi targets and non-structural gene RNAi targets. The inhibitory efficacy of each lhRNA expression cassette was quantified by flow cytometry. As shown in **Figure 2B**, we chose 40% inhibition efficiency as the selection standard. The inhibitory efficacy of each lhRNA expression cassette against HIV genes is shown in Supplementary Table 4. The Gag-Vpu-lhRNA and Vpu-Gag-lhRNA expression cassettes were chosen because they inhibited Gag-eGFP expression. The Vpu-Gag-lhRNA and Gag-Vpu-lhRNA encoding cassettes were chosen because they inhibited Vpu-eGFP expression. The Tat-Env-lhRNA encoding cassette was chosen because it inhibited Tat-eGFP expression. The Env-Tat-lhRNA and Tat-Env-lhRNA encoding cassettes were chosen because they inhibited Env-eGFP expression. The Vpu-Gag and Tat-Env lhRNA expression cassettes were chosen for the dlhRNA expression cassette because they inhibited each of the target HIV genes. Two dlhRNA expression cassettes, abbreviated TEVG and VGTE (TEVG: Tat-Env lhRNA followed by Vpu-Gag lhRNA; VGTE: Vpu-Gag lhRNA followed by Tat-Env lhRNA), were derived from the combinations of lhRNA expression cassettes chosen previously. As shown in **Figure 2C**, VGTE inhibited expression of Gag-eGFP (36.5% vs. 18.9%) and Vpu-eGFP (59% vs. 14.3%) more effectively than TEVG, while TEVG inhibited expression of Tat-eGFP (55.4% vs. 37.5%) and Env-eGFP (36.6% vs. 22.5%) more effectively than VGTE. The difference between Gag-eGFP and Vpu-eGFP inhibition by VGTE and TEVG was significant, while the difference between Tat-eGFP and Env-eGFP inhibition by VGTE and TEVG was not significant (*p* > 0.05). Based on these results, VGTE was chosen as the dlhRNA expression cassette in the following experiments.

**FIGURE 3:**
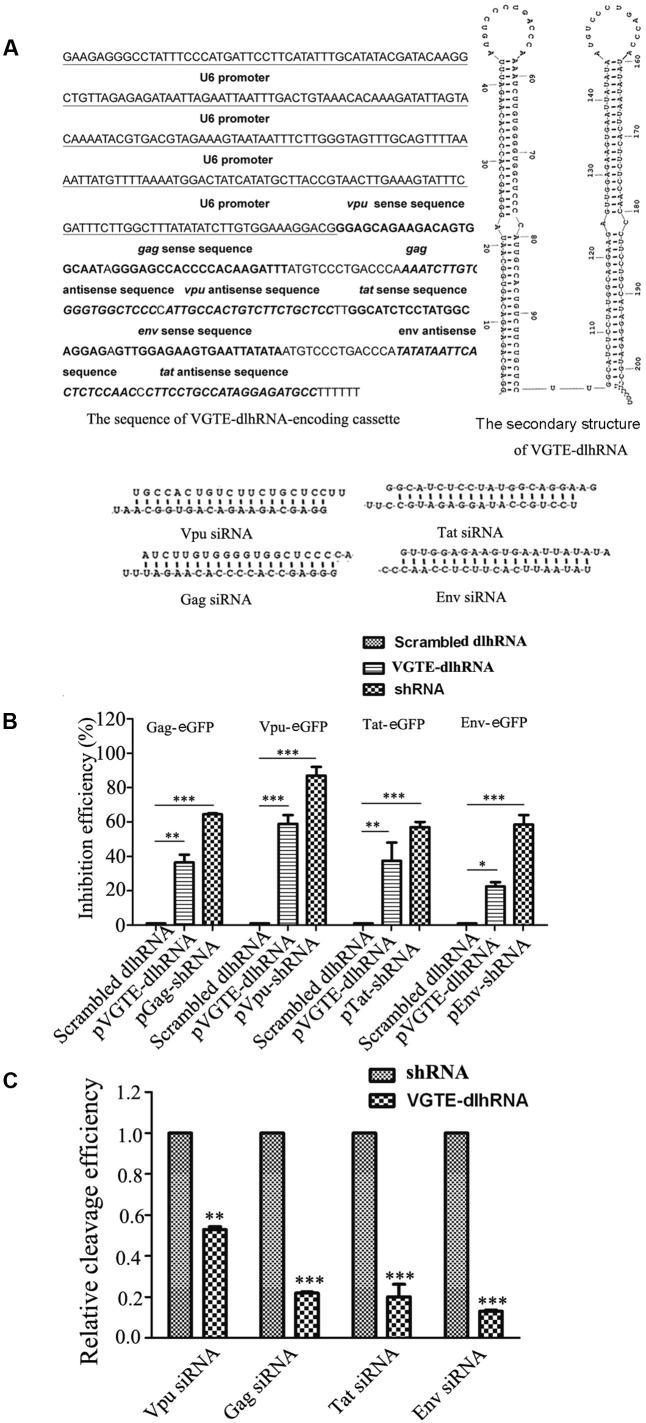
Functional analyses of the VGTE-dlhRNA expression cassette. **(A)** Overview of the VGTE-dlhRNA expression cassette, including the sequence information of the cassette, RNA secondary structure, and four cleaved siRNAs. **(B)** Inhibitory efficacy of VGTE-dlhRNA and shRNAs. The reduction in HIV gene/eGFP fusion protein expression was taken as the inhibitory efficacy of RNAi. ^∗^*p* < 0.05, ^∗∗^*p* < 0.01, ^∗∗∗^*p* < 0.001, compared with the scrambled dlhRNA-treated control. **(C)** Relative cleavage efficacy of siRNAs. HEK293T cells were transfected with VGTE-dlhRNA or the corresponding shRNA plasmid. After 2 days, total RNA was extracted, and siRNA was quantified by stem–loop RT-PCR. The results were normalized by the corresponding shRNA. Compared with the corresponding shRNA, the relative abundance of each siRNA generated from VGTE-dlhRNA was 0.53 (Vpu), 0.22 (Gag), 0.20 (Tat), and 0.17 (Env). ^∗∗^*p* < 0.01, ^∗∗∗^*p* < 0.001, VGTE–dlhRNA vs. corresponding shRNA treated groups. All experiments were performed more than three times.

Detailed information about the VGTE-dlhRNA expression cassette is shown in **Figure 3A**, including the nucleotide sequence of the dlhRNA expression cassette, the secondary structure of the double-long hairpin RNA, and the four different siRNAs generated after cleavage. The RNAi efficacy of VGTE-dlhRNA compared with each shRNA is summarized in **Figure 3B**. The efficacy of VGTE-dlhRNA was not as high as that of each corresponding shRNA (Gag: 36.5% vs. 64.3%, Vpu: 59% vs. 88%, Tat: 37.5% vs. 58%, Env: 22.5% vs. 60.7%). These results suggest that the VGTE-dlhRNA expression cassette was constructed successfully and inhibited HIV-1 gene expression. The difference in inhibitory efficacy may have been due to reduced siRNA generation by the dlhRNA. siRNA generation by VGTE-dlhRNA in HEK293T cells was quantified by qRT-PCR and compared with that of each shRNA. As shown in **Figure 3C**, *vpu/gag/tat/env* siRNAs were generated by the VGTE-dlhRNA expression cassette, but the rate of siRNA generation was reduced compared with that of each corresponding shRNA (Vpu: 53%, Gag: 22%, Tat: 20%, Env: 17%; data were normalized by the corresponding shRNA group).

### Construction and Functional Assays of the VGTE-dlhRNA Expression Cassette Delivering Lentiviral Vector and VGTE-TZM-bl Cells Stably Expressing VGTE-dlhRNA

**FIGURE 4:**
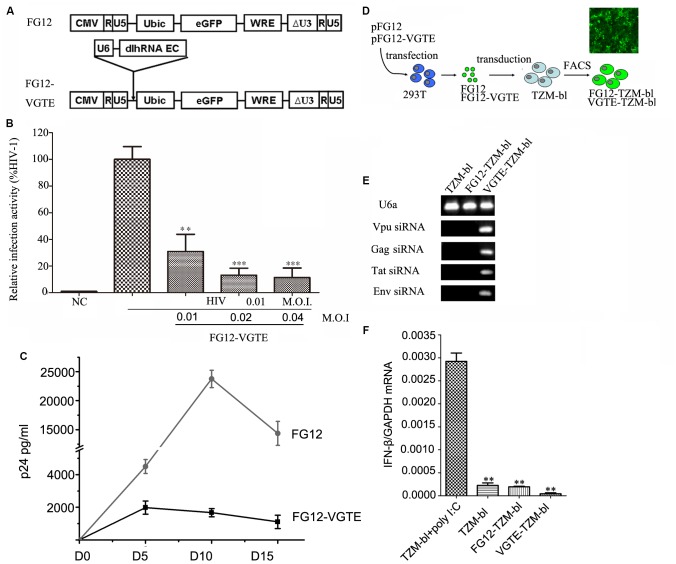
Construction and functional assay of VGTE-dlhRNA expressing lentiviral vector FG12-VGTE and VGTE-TZM-bl cells stably expressing VGTE-dlhRNA. **(A)** Overview of VGTE-dlhRNA expressing lentiviral vector VGTE-FG12 compared with empty lentiviral vector FG12. “U6 dlhRNA EC” represented the VGTE-dlhRNA-encoding cassette. “eGFP” represented the eGFP expressing element as a selectable marker. **(B)** FG12-VGTE inhibited replication of HIV-1 in TZM-bl cells. TZM-bl cells were challenged with 0.01 M.O.I. HIV-1 and treated with 0.01/0.02/0.04 M.O.I. FG12-VGTE or FG12. Finally, cells were harvested and luciferase activity was measured. NC represented the non-infected and non-treated group, which served as a negative control group. The group only infected with HIV was taken as a standard (100%), which was used to normalize the other groups. ^∗∗^*p* < 0.01, ^∗∗∗^*p* < 0.001, compared with the group only infected with HIV-1. **(C)** FG12-VGTE inhibited replication of HIV-1 in CD4+ T cells. CD4+ T cells were transduced with FG12-VGTE or FG12 after activation with PHA for 2 days, and then were infected with 0.01 M.O.I HIV-1. Culture supernatants harvested on days 0, 5, 10, and 15 after HIV infection were tested for released virus levels by p24 ELISA. **(D)** Flowchart of VGTE-TZM-bl and FG12-TZM-bl cell generation. FG12-VGTE or FG12 was packaged and harvested in HEK293T cells, after which TZM-bl cells were transducted by FG12-VGTE or FG12. After 3 days, GFP-positive cells were selected by fluorescence-activated cell sorting. GFP-positive cells were VGTE-TZM-bl cells that stably expressed VGTE-dlhRNA or FG12-TZM-bl cells that were empty lentiviral vector integrated control TZM-bl cells. **(E)** Four siRNAs were expressed in VGTE-TZM-bl cells. Total RNA was extracted from TZM-bl/FG12-TZM-bl/VGTE-TZM-bl cells and analyzed by stem–loop PCR. U6a small RNA was used as an endogenous control. Vpu/Gag/Tat/Env siRNAs were expressed in VGTE-TZM-bl cells, but not expressed in TZM-bl or FG12-TZM-bl cells. **(F)** Potential induction of the IFN response in VGTE-TZM-bl cells. IFN induction was assessed by measuring the IFN-β mRNA concentration in total RNA. TZM-bl cells treated with poly I:C served as a positive control. Untreated TZM-bl cells served as a negative control. The mean normalized ratios of IFN-β:GAPDH were determined by qRT-PCR. ^∗∗^*p* < 0.01, compared with poly I:C-treated TZM-bl cells.

After the VGTE dlhRNA expression cassette was constructed successfully, it was sub-cloned into lentiviral vector FG12 to construct FG12-VGTE. The structure of FG12-VGTE included a VGTE dlhRNA expression cassette and an eGFP expression element, as shown in **Figure 4A**. FG12-VGTE and the negative control FG12 lentiviral particles were packaged in HEK293T cells and harvested. The transient inhibitory efficacy of FG12-VGTE against HIV-1 replication is shown in **Figure 4B**. FG12-VGTE at a M.O.I. of 0.01, 0.02, and 0.04 reduced HIV NL4.3 replication by 69.1, 86.9, and 88.6%, respectively, in TZM-bl cells.

To evaluate the inhibitory effect of FG12-VGTE in CD4+ T cells, CD4+ T cells were transduced with FG12-VGTE or FG12 after activation with PHA for 2 days, and then after 2 days the cultures were infected with HIV-1 NL4-3. Culture supernatants harvested on days 0, 5, 10, 15 after HIV infection were tested for released virus levels by p24 ELISA. As shown in **Figure 4C**, HIV-1 replication showed a peak on day 10 and declined by day 15 in FG12 transducing CD4+ T cells. Compared with the FG-12 group, the replication of HIV was repressed significantly in FG12-VGTE transducing CD4+ T cells.

To obtain HIV-1 susceptible cells that stably expressed VGTE-dlhRNA, TZM-bl cells were transduced by FG12-VGTE and sorted via FACS at 3 days post-transduction as shown in **Figure 4D**. Highly eGFP-positive cells were collected, and the percentage of GFP-positive cells was greater than 98%. The GFP-positive cells were designated VGTE-TZM-bl cells. Fluorescence images were taken 2 days after sorting. Negative control FG12-TZM-bl cells were obtained similarly by transduction with FG12. The expression levels of four siRNAs were detected by stem–loop RT-PCR. As shown in **Figures 4E,F**, four siRNAs were detected in the VGTE-TZM-bl cells, but not in the TZM-bl or FG12-TZM-bl cells, as expected. IFN-β was also detected in the VGTE-TZM-bl cells. These results suggest that VGTE dlhRNA expression did not induce a detectable IFN response.

### HIV Replication Assay in VGTE-TZM-bl Cells

**FIGURE 5:**
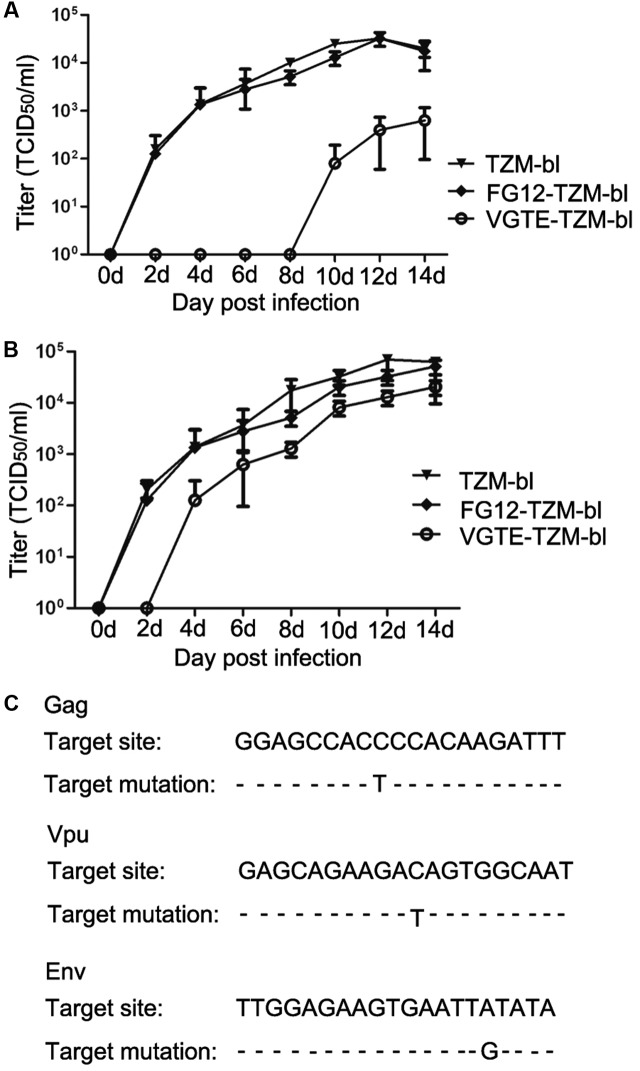
HIV-1 escaping from inhibition by stable VGTE-dlhRNA expression. **(A)** Replication kinetics of HIV-1 NL4-3 in TZM-bl, FG12-TZM-bl, and VGTE-TZM-bl cells. Cells were challenged by 0.01 M.O.I. HIV-1 NL4-3, and cell culture supernatants were collected for the TCID_50_ assay. Replication of NL4-3 was not detected in VGTE-TZM-bl cells in the first 8 days, but NL4-3 replicated well in TZM-bl and FG12-TZM-bl cells. **(B)** Replication kinetics of escaping HIV-1 NL4-3 in TZM-bl, FG12-TZM-bl and VGTE-TZM-bl cells. The assay process was similar to the former assay. The challenge virus mutated into escaping HIV-1 in VGTE-TZM-bl cells. Unlike NL4-3, replication of escaping HIV-1 was detected 4 days after infection challenge. **(C)** Mutant RNAi targets. Escaping HIV-1 from VGTE-TZM-bl cells was sequenced. Mutant forms of Gag, Vpu, and Env emerged.

After obtaining VGTE-TZM-bl cells with stable VGTE dlhRNA expression, we challenged these cells with HIV-1 NL4-3. TZM-bl and FG12-TZM-bl cells were also challenged as control groups. As shown in **Figure 5A**, the replication kinetics of NL4-3 in TZM-bl and FG12-TZM-bl cells were not significantly different. In VGTE-TZM-bl cells, NL4-3 replication was inhibited completely for the first 8 days, after which the replication rate increased. In comparison with the TZM-bl and FG12-TZM-bl groups, the HIV replication rate of the VGTE-TZM-bl cells was decreased by two orders of magnitude 14 days post-infection. In order to assess virus survival in VGTE-TZM-bl cells, viruses were collected from the culture supernatants 14 days post-infection and used to re-infect VGTE-TZM-bl cells. As shown in **Figure 5B**, HIV replication in VGTE-TZM-bl cells was only inhibited completely for the first 2 days, after which replication recovered quickly. In contrast to the previous challenge, the viral replication rate in the VGTE-TZM-bl cells was nearly equal to that of the two control groups. These results suggest that long-term combinatorial RNAi with VGTE dlhRNA did not inhibit replication of HIV-1 because mutant versions of target mRNAs emerged under the combinatorial RNAi condition. To assess this possibility, proviral DNA was isolated from VGTE-TZM-bl cells and sequenced. As shown in **Figure 5C**, nucleotide mutant versions of Vpu, Gag and Env were found, but no mutation in Tat was identified.

## Discussion

RNAi based approaches represent powerful gene therapy strategies for patients infected with HIV-1. The error-prone replication mechanism of HIV makes the virus particularly susceptible to RNAi strategies based on two principles: (i) utilization of several siRNAs against different targets; and (ii) selection of RNAi targets corresponding to conserved regions in the viral genome. In this study, we followed both of these principles. Four selected RNAi targets were located in the conserved regions of different viral genes, and an optimized dlhRNA expression cassette that encoded four siRNAs against those targets was constructed successfully. It has been reported that utilizing at least four siRNAs can repress HIV replication and avoid escape mutations (Leonard and Schaffer, 2005; ter Brake and Berkhout, 2005). Therefore, viral genes *gag, tat, vpu*, and *env* were selected. All of the targets are located on relatively conserved regions of the viral genome. The LTR region of HIV is highly conserved and contains numerous potential RNAi targets. However, siRNAs against the LTR region were not used in this study because of their potential to disrupt lentiviral vector FG12. First, four shRNA expression cassettes were constructed and tested. The RNAi efficacy assay showed that all shRNA expression cassettes decreased viral gene expression more than 50%, while the inhibitory efficacy of Vpu-shRNA was nearly 90%. In our study, we applied a dlhRNA expression cassette to generate four different siRNAs. The arrangement of siRNAs can affect RNAi efficacy, so a series of lhRNA/dlhRNA expression cassettes was constructed and tested. An optimized dlhRNA expression cassette, designated VGTE-dlhRNA, was constructed. VGTE-dlhRNA generated four different siRNAs from one RNA precursor and had a reasonable inhibitory effect on viral gene expression. The inhibitory efficacy of VGTE-dlhRNA was lower than that of each shRNA expression cassette. Next, the lentiviral vector that was used to deliver the VGTE-dlhRNA expression cassette, which was designated FG12-VGTE, was constructed. Under transient expression of VGTE-dlhRNA, HIV-1 NL4-3 replication was inhibited as expected.

To study the long-term inhibitory effect of VGTE-dlhRNA, FACS was used to sort the HIV-susceptible VGTE-TZM-bl cells with stable VGTE-dlhRNA expression. Four siRNAs were detected in the VGTE-TZM-bl cells. No non-specific IFN pathway activation was detected in the VGTE-TZM-bl cells, as expected. However, the long-term inhibitory effect of VGTE-dlhRNA was not as strong as expected. Although HIV-1 NL-43 replication was inhibited successfully by VGTE-dlhRNA during the first 6 days of the experiment, viral replication eventually recovered as nucleotide mutants of the RNAi targets emerged. The lack of long-term inhibition might have been due to the relatively small amount of each siRNA generated by VGTE-dlhRNA in comparison with that generated by each shRNA. The ability of combinatorial siRNAs to repress the emergence of HIV mutations is controversial. Shah suggested that HIV-1 can escape from the effects of combinatorial RNAi by selecting mutations in regions of the viral genome other than the RNAi targets (Shah et al., 2012). However, Ben and Atze suggested that the mutation “pseudomorph” came from HIV-1 quasispecies replicated in mixed cell cultures of protected and unprotected cells (Berkhout and Das, 2012). Some reports show that even a single point mutation in a target sequence can allow HIV-1 to escape RNAi (Pusch et al., 2003; Senserrich et al., 2008), which happened in our study. HIV-1 RNAs have natural resistance to RNAi because viral transcripts in complexes with proteins have limited accessibility. Viral RNA is packaged into a nucleoprotein complex at the pre-integration stage of infection and coated with gag precursor at the late stage of replication, so there is a very short window of time in which viral RNAs can be attacked by RNAi.

HIV is an error-prone virus; therefore, RNAi against HIV is like “shooting moving targets,” and combinatorial siRNA is like “shooting multiple targets.” The success of each method depends on the RNAi efficacy and mutation rate. It is better to choose RNAi targets that do not change rapidly, just as we did in this study. However, after the long-term inhibition test of our combinatorial siRNAs, viral replication recovered and mutations emerged. Our failure may be partly due to the low siRNA generation rate of the VGTE-dlhRNA expression cassette in comparison with that of each independent shRNA expression cassette. RNAi targets should be as stable as possible. Some host factors that are essential for HIV-1 replication, such as CCR5, the co-receptor of HIV-1, are ideal RNAi targets. It has been reported that the “Berlin patient” was cured of HIV by transplanting hematopoietic stem cell from an individual homozygous for the detal32 CCR5 deletion (Hutter et al., 2009; Allers et al., 2011). Several groups have achieved some experimental successes; Choi et al. (2015) and Spanevello et al. (2016) used combinatorial siRNAs against CCR5 and viral genes to repress HIV-1 in cell lines and PBMCs successfully. Although we did not identify a successful long-term combinatorial siRNA against HIV-1, several important findings emerged from this study: (i) RNAi target selection among viral genes and host genes essential for HIV is a very important process; (ii) the long RNA expression cassette should be optimized and improved to generate more siRNAs; (iii) a good RNAi delivery vector is important. Our findings demonstrate the therapeutic potential of stably expressed combinatorial siRNAs generated from a single dlhRNA precursor against error-prone viruses like HIV-1.

## Author Contributions

CL and XK helped design the experiments. CL and ZL carried out all the experiments and statistical analysis. All authors drafted, read and approved the final manuscript. All authors agree to be accountable for all aspects of the work in ensuring that questions related to the accuracy or integrity of any part of the work are appropriately investigated and resolved.

## Conflict of Interest Statement

The authors declare that the research was conducted in the absence of any commercial or financial relationships that could be construed as a potential conflict of interest.

## Footnotes

1 http://www.hiv.lanl.gov

2 http://sivirus.rnai.jp/HIV/

## References

[B1] Allers K. Hutter G. Hofmann J. Loddenkemper C. Rieger K. Thiel E. (2011). Evidence for the cure of HIV infection by CCR5Delta32/Delta32 stem cell transplantation. *Blood* 117 2791–2799. 10.1182/blood-2010-09-309591 21148083

[B2] Berkhout B. Das A. T. (2012). HIV-1 escape from RNAi antivirals: Yet another Houdini action? *Mol. Ther. Nucleic Acids.* 1 e26. 10.1038/mtna.2012.22 PMC3390223 23344078

[B3] Brummelkamp T. R. Bernards R. Agami R. (2002). A system for stable expression of short interfering RNAs in mammalian cells. *Science* 296 550–553. 10.1126/science.1068999 11910072

[B4] Castanotto D. Li H. Rossi J. J. (2002). Functional siRNA expression from transfected PCR products. *RNA* 8 1454–1460. 10.1017/S1355838202021362 12458798 PMC1370351

[B5] Chen C. Ridzon D. A. Broomer A. J. Zhou Z. Lee D. H. Nguyen J. T. (2005). Real-time quantification of microRNAs by stem-loop RT-PCR. *Nucleic Acids Res.* 33 e179 10.1093/nar/gni178 PMC1292995 16314309

[B6] Choi J. G. Bharaj P. Abraham S. Ma H. Yi G. Ye C. (2015). Multiplexing seven miRNA-Based shRNAs to suppress HIV replication. *Mol. Ther.* 23 310–320. 10.1038/mt.2014.205 25358251 PMC4445613

[B7] Deng Y. Wang C. C. Choy K. W. Du Q. Chen J. Wang Q. (2014). Therapeutic potentials of gene silencing by RNA interference: principles, challenges, and new strategies. *Gene* 538 217–227. 10.1016/j.gene.2013.12.019 24406620

[B8] Haasnoot J. Westerhout E. M. Berkhout B. (2007). RNA interference against viruses: strike and counterstrike. *Nat. Biotechnol.* 25 1435–1443. 10.1038/nbt1369 18066040 PMC7096910

[B9] Hutter G. Nowak D. Mossner M. Ganepola S. Mussig A. Allers K. (2009). Long-term control of HIV by CCR5 Delta32/Delta32 stem-cell transplantation. *N. Engl. J. Med.* 360 692–698. 10.1056/NEJMoa0802905 19213682

[B10] Kong X. West J. T. Zhang H. Shea D. M. M’Soka T. J. Wood C. (2008). The human immunodeficiency virus type 1 envelope confers higher rates of replicative fitness to perinatally transmitted viruses than to nontransmitted viruses. *J. Virol.* 82 11609–11618. 10.1128/JVI.00952-08 18786994 PMC2583653

[B11] Leonard J. N. Schaffer D. V. (2005). Computational design of antiviral RNA interference strategies that resist human immunodeficiency virus escape. *J. Virol.* 79 1645–1654. 10.1128/JVI.79.3.1645-1654.2005 15650190 PMC544124

[B12] Liang Z. Guo Z. Wang X. Kong X. Liu C. (2012). Two retroviruses packaged in one cell line can combined inhibit the replication of HIV-1 in TZM-bl cells. *Virol. Sin.* 27 339–344. 10.1007/s12250-012-3263-8 23188559 PMC8218129

[B13] Liu Y. P. Haasnoot J. Berkhout B. (2007). Design of extended short hairpin RNAs for HIV-1 inhibition. *Nucleic Acids Res.* 35 5683–5693. 10.1093/nar/gkm596 17715143 PMC2034457

[B14] Liu Y. P. von Eije K. J. Schopman N. C. Westerink J. T. ter Brake O. Haasnoot J. (2009). Combinatorial RNAi against HIV-1 using extended short hairpin RNAs. *Mol. Ther.* 17 1712–1723. 10.1038/mt.2009.176 19672247 PMC2835024

[B15] Margolis D. M. Garcia J. V. Hazuda D. J. Haynes B. F. (2016). Latency reversal and viral clearance to cure HIV-1. *Science* 353:aaf6517 10.1126/science.aaf6517 PMC5021637 27463679

[B16] McIntyre G. J. Groneman J. L. Yu Y. H. Jaramillo A. Shen S. Applegate T. L. (2009). 96 shRNAs designed for maximal coverage of HIV-1 variants. *Retrovirology* 6:55 10.1186/1742-4690-6-55 PMC2698899 19497094

[B17] Meister G. Tuschl T. (2004). Mechanisms of gene silencing by double-stranded RNA. *Nature* 431 343–349. 10.1038/nature02873 15372041

[B18] Mollaie H. R. Monavari S. H. Arabzadeh S. A. Shamsi-Shahrabadi M. Fazlalipour M. Afshar R. M. (2013). RNAi and miRNA in viral infections and cancers. *Asian Pac. J. Cancer Prev.* 14 7045–7056. 10.7314/APJCP.2013.14.12.7045 24460249

[B19] Naito Y. Nohtomi K. Onogi T. Uenishi R. Ui-Tei K. Saigo K. (2007). Optimal design and validation of antiviral siRNA for targeting HIV-1. *Retrovirology* 4:80 10.1186/1742-4690-4-80 PMC2204037 17996047

[B20] Paddison P. J. Caudy A. A. Bernstein E. Hannon G. J. Conklin D. S. (2002). Short hairpin RNAs (shRNAs) induce sequence-specific silencing in mammalian cells. *Genes Dev.* 16 948–958. 10.1101/gad.981002 11959843 PMC152352

[B21] Passaes C. P. Saez-Cirion A. (2014). HIV cure research: advances and prospects. *Virology* 45 340–352. 10.1016/j.virol.2014.02.021 24636252

[B22] Pennings P. S. (2013). HIV drug resistance: problems and perspectives. *Infect Dis. Rep.* 5(Suppl. 1):e5 10.4081/idr.2013.s1.e5 PMC3892620 24470969

[B23] Philippidis A. (2013). Gene therapy briefs. *Hum. Gene Ther.* 24 565–567. 10.1089/hum.2013.250 23777463 PMC3689177

[B24] Pusch O. Boden D. Silbermann R. Lee F. Tucker L. Ramratnam B. (2003). Nucleotide sequence homology requirements of HIV-1-specific short hairpin RNA. *Nucleic Acids Res.* 31 6444–6449. 10.1093/nar/gkg876 14602902 PMC275570

[B25] Saayman S. Arbuthnot P. Weinberg M. S. (2010). Deriving four functional anti-HIV siRNAs from a single Pol III-generated transcript comprising two adjacent long hairpin RNA precursors. *Nucleic Acids Res.* 38 6652–6663. 10.1093/nar/gkq460 20525791 PMC2965221

[B26] Senserrich J. Pauls E. Armand-Ugon M. Clotet-Codina I. Moncunill G. Clotet B. (2008). HIV-1 resistance to the anti-HIV activity of a shRNA targeting a dual-coding region. *Virology* 372 421–429. 10.1016/j.virol.2007.10.045 18068205

[B27] Shah P. S. Pham N. P. Schaffer D. V. (2012). HIV develops indirect cross-resistance to combinatorial RNAi targeting two distinct and spatially distant sites. *Mol. Ther.* 20 840–848. 10.1038/mt.2012.3 22294151 PMC3321590

[B28] Spanevello F. Calistri A. Del Vecchio C. Mantelli B. Frasson C. Basso G. (2016). Development of lentiviral vectors simultaneously expressing multiple siRNAs against CCR5, vif and tat/rev genes for an HIV-1 gene therapy approach. *Mol. Ther. Nucleic Acids* 5 e312 10.1038/mtna.2016.24 PMC5014525 27093170

[B29] ter Brake O. Berkhout B. (2005). A novel approach for inhibition of HIV-1 by RNA interference: counteracting viral escape with a second generation of siRNAs. *J. RNAi Gene Silencing* 1 56–65. 19771206 PMC2737200

[B30] von Eije K. J. ter Brake O. Berkhout B. (2008). Human immunodeficiency virus type 1 escape is restricted when conserved genome sequences are targeted by RNA interference. *J. Virol.* 82 2895–2903. 10.1128/JVI.02035-07 18077712 PMC2258968

[B31] Westerhout E. M. Ooms M. Vink M. Das A. T. Berkhout B. (2005). HIV-1 can escape from RNA interference by evolving an alternative structure in its RNA genome. *Nucleic Acids Res.* 33 796–804. 10.1093/nar/gki220 15687388 PMC548362

[B32] Woods E. A. Foisy M. M. (2014). Antiretroviral-related alopecia in HIV-infected patients: a review of the literature. *Ann. Pharmacother.* 48 1187–1193. 10.1177/1060028014540451 24944240

