# Supplementary material for: Efficacy Analysis of Combinatorial siRNAs against HIV Derived from One Double Hairpin RNA Precursor
Source: Front Microbiol. 2017 Aug 29;8:1651. doi: 10.3389/fmicb.2017.01651 (PMC5581867; doi:10.3389/fmicb.2017.01651)
Supplement: Supplementary file 1 [file Table_1.DOC]

Supplementary Material

# Efficacy Analysis of Combinatorial siRNAs against HIV Derived from One Double Hairpin RNA Precursor

Chang Liu, Zhipin Liang, Xiaohong Kong*

*** Correspondence:** Xiaohong Kong: kongxh@nankai.edu.cn

# Supplementary Tables

**Supplementary Table 1.** Sequences of the primers used to construct HIV/EGFP fusion protein expression plamids. Primer names, nucleotide sequences, and nucleic acid lengths are listed in the following table. Underlined sequences represent restriction endonuclease sites used in the construction.

| Primer | 5′-3′ Sequence | Length (nt) |
| --- | --- | --- |
| Gag F | CGGAATTCCGATGGGATCAGAAGAACTTAGATC | 33 |
| Gag R | CGGGATCCCGCCCTGCATGCACTGGATGC | 29 |
| Tat F | CGGAATTCCGATGGAGCCAGTAGATCCTAG | 30 |
| Tat R | CGGGATCCCGCTTTGATAGAGAAGCTTGATGAG | 33 |
| Vpu F | CGGAATTCCGATGACGCAACCTATACCAATAGTAGC | 36 |
| Vpu R | CGGGATCCCGCAGATCATCAACATCCCAAGGAGC | 34 |
| Env F | CGGAATTCCGATGTGGCAGGAAGTAGGAAAAGC | 33 |
| Env R | CGGGATCCCGTGCTGCTCCCAAGAACCCAAGG | 32 |

**Supplementary Table 2.** Sequences of the primers used to construct the hairpin RNA expression cassettes. Primer names, nucleotide sequences, and nucleic acid lengths are listed in the following table. Underlined and italics sequences represent siRNA-encoding sequences.

| Primer | 5′-3′ Sequence | Length (nt) |
| --- | --- | --- |
| hU6 F primer | AAGATCTGGGCAGGAAGAGGG | 21 |
| Gag-shRNA-R | AAAAAA*GGGAGCCACCCCACAAGATTT*TGGGTCAGGAAATCTTGTGGGGTGGCTCCCGACTCTCGTCCTTTCCACAAG | 78 |
| Tat-shRNA-R | AAAAAA*GGCATCTCCTATGGCAGGAAG*TGGGTCAGGCTTCCTGCCATAGGAGATGCCGACTCTCGTCCTTTCCACAAG | 78 |
| Vpu-shRNA-R | AAAAAA*GGAGCAGAAGACAGTGGCAAT*TGGGTCAGGATTGCCACTGTCTTCTGCTCCGACTCTCGTCCTTTCCACAAG | 78 |
| Env-shRNA-R | AAAAAA*GTTGGAGAAGTGAATTATATA*TGGGTCAGGTATATAATTCACTTCTCCAACGACTCTCGTCCTTTCCACAAG | 78 |
| Scr-shRNA-R | AAAAAA*GATTGATGTAAAGGATGATTA*TGGGTCAGGTAATCATCCTTTACATCAATCCGACTCTCGTCCTTTCCACAAG | 78 |
| Gag-Tat-lhRNA-R1 | CTGGGTCAGGGACATCTTCCTGCCATAGGAGATGCCTAAATCTTGTGGGGTGGCTCCCGACTCTCGTCCTTTCCACAAG | 79 |
| Gag-Tat-lhRNA-R2 | AAAAAA*GGGAGCCACCCCACAAGATTT*G*GGCATCTCCTATGGCAGGAAG*ACGTCTGGGTCAGGGACATCTTCC | 73 |
| Tat-Gag-lhRNA-R1 | CTGGGTCAGGGACATAAATCTTGTGGGGTGGCTCCCTCTTCCTGCCATAGGAGATGCCGACTCTCGTCCTTTCCACAAG | 79 |
| Tat-Gag-lhRNA-R2 | AAAAAA*GGCATCTCCTATGGCAGGAAG*G*GGGAGCCACCCCACAAGATTT*ACGTCTGGGTCAGGGACATAAATC | 73 |
| Env-Vpu-lhRNA-R1 | CTGGGTCAGGGACATATTGCCACTGTCTTCTGCTCCTTATATAATTCACTTCTCCAACGACTCTCGTCCTTTCCACAAG | 79 |
| Env-Vpu-lhRNA-R2 | AAAAAA*GTTGGAGAAGTGAATTATATA*G*GGAGCAGAAGACAGTGGCAAT*ACGTCTGGGTCAGGGACATATTGC | 73 |
| Vpu-Env-lhRNA-R1 | CTGGGTCAGGGACATTATATAATTCACTTCTCCAACTATTGCCACTGTCTTCTGCTCCGACTCTCGTCCTTTCCACAAG | 79 |
| Vpu-Env-lhRNA-R2 | AAAAAA*GGAGCAGAAGACAGTGGCAAT*G*GTTGGAGAAGTGAATTATATA*ACGTCTGGGTCAGGGACATTATATAATTC | 78 |
| Gag-Vpu-lhRNA-R1 | CTGGGTCAGGGACATATTGCCACTGTCTTCTGCTCCTAAATCTTGTGGGGTGGCTCCCGACTCTCGTCCTTTCCACAAG | 79 |
| Gag-Vpu-lhRNA-R2 | AAAAAA*GGGAGCCACCCCACAAGATTT*G*GGAGCAGAAGACAGTGGCAAT*ACGTCTGGGTCAGGGACATATTGC | 73 |
| Vpu-Gag-lhRNA-R1 | 5'-CTGGGTCAGGGACATAAATCTTGTGGGGTGGCTCCCTATTGCCACTGTCTTCTGCTCCGACTCTCGTCCTTTCCACAAG-3' | 79 |
| Vpu-Gag-lhRNA-R2 | AAAAAA*GGAGCAGAAGACAGTGGCAAT*G*GGGAGCCACCCCACAAGATTT*ACGTCTGGGTCAGGGACATAAATC | 73 |
| Env-Tat-lhRNA-R1 | CTGGGTCAGGGACATCTTCCTGCCATAGGAGATGCCTTATATAATTCACTTCTCCAACGACTCTCGTCCTTTCCACAAG | 79 |
| Env-Tat-lhRNA-R2 | AAAAAA*GTTGGAGAAGTGAATTATATA*G*GGCATCTCCTATGGCAGGAAG*ACGTCTGGGTCAGGGACATCTTCC | 73 |
| Tat-Env-lhRNA-R1 | CTGGGTCAGGGACATTATATAATTCACTTCTCCAACTCTTCCTGCCATAGGAGATGCCGACTCTCGTCCTTTCCACAAG | 79 |
| Tat-Env-lhRNA-R2 | AAAAAA*GGCATCTCCTATGGCAGGAAG*G*GTTGGAGAAGTGAATTATATA*ACGTCTGGGTCAGGGACATTATATAATTC | 78 |
| Scr-lhRNA-R1 | CTGGGTCAGGGACATACACTTCCCGTCTTTGCTTGCTTAATCATCCTTTACATCAATCGACTCTCGTCCTTTCCACAAG | 79 |
| Scr-lhRNA-R2 | AAAAAA*GATTGATGTAAAGGATGATTA*G*GCAAGCAAAGACGGGAAGTGT*ACGTCTGGGTCAGGGACATACAC | 72 |
| TEVG-dlhRNA-R1 | CTGGGTCAGGGACATAAATCTTGTGGGGTGGCTCCCTATTGCCACTGTCTTCTGCTCCAA*GGCATCTCCTATGGCAGGAAG* | 81 |
| TEVG-dlhRNA-R2 | AAAAAA*GGAGCAGAAGACAGTGGCAATGGGAGCCACCCCACAAGATTT*TGGGTCAGGGACAT | 62 |
| VGTE-dlhRNA-R1 | CTGGGTCAGGGACATTATATAATTCACTTCTCCAACTCTTCCTGCCATAGGAGATGCCAA*GGAGCAGAAGACAGTGGCAAT* | 81 |
| VGTE-dlhRNA-R2 | AAAAAA*GGCATCTCCTATGGCAGGAAGGTTGGAGAAGTGAATTATATA*TGGGTCAGGGACAT | 62 |
| Scr-dlhRNA-R1 | CTGGGTCAGGGACATATGTAACGCTTGTGCGGCTGCTTGATGTCTCGCAACGCTGCACAAGATTGATGTAAAGGATGATTA | 81 |
| Scr-dlhRNA-R2 | AAAAAA*GTGCAGCGTTGCGAGACATCAGCAGCCGCACAAGCGTTACAT*TGGGTCAGGGACAT | 62 |

**Supplementary Table 3.** Sequences of the primers used in the RT-PCR assay. Primer names, nucleotide sequences, and nucleic acid lengths are listed in the following table.

| Primer | 5′-3′ Sequence | Length (nt) |
| --- | --- | --- |
| U6a-siRNA-RT | GTCGTATCCAGTGCAGGGTCCGAGGTATTCGCACTGGATACGACAAAATATGGAAC | 56 |
| Gag-siRNA -RT | GTCGTATCCAGTGCAGGGTCCGAGGTATTCGCACTGGATACGACGGGAGC | 50 |
| Tat-siRNA -RT | GTCGTATCCAGTGCAGGGTCCGAGGTATTCGCACTGGATACGACGGCATCT | 51 |
| Vpu-siRNA -RT | GTCGTATCCAGTGCAGGGTCCGAGGTATTCGCACTGGATACGACGGAGCAG | 51 |
| Env-siRNA -RT | GTCGTATCCAGTGCAGGGTCCGAGGTATTCGCACTGGATACGACGTTGGA | 50 |
| U6a-siRNA -F | TGCGGGTGCTCGCTTCGGCAGC | 22 |
| Universal-R | CAGTGCAGGGTCCGAGGT | 18 |
| Gag-siRNA -F | GGCGAAATCTTGTGGGGTGG | 20 |
| Tat-siRNA -F | CGTGGACTTCCTGCCATAGG | 20 |
| Vpu-siRNA -F | GCCACGATTGCCACTGTCTTC | 21 |
| Env-siRNA -F | ACCGCCCGTATATAATTCACTTCTC | 25 |
| IFN-β-F | TCCAAATTGCTCTCCTGTTGTGCT | 24 |
| IFN-β-R | CCACAGGAGCTTCTGACACTGAAAA | 25 |
| GAPDH-F | TGCACCACCAACTGCTTAGC | 20 |
| GAPDH-R | GGCATGGACTGTGGTCATGAG | 21 |

**Supplementary Table 4. The inhibitory efficacy of lhRNA-expression cassettes against expression of HIV gene EGFP fusion proteins.**

|  | Gag-EGPF | Tat-EGFP | Vpu-EGFP | Env-EGFP |
| --- | --- | --- | --- | --- |
| Gag-Tat-lhRNA | 38% | 18% | n/a | n/a |
| Tat-Gag-lhRNA | 16% | 22% | n/a | n/a |
| Gag-Vpu-lhRNA | 51% | n/a | 36% | n/a |
| Vpu-Gag-lhRNA | 40% | n/a | 75% | n/a |
| Env-Tat-lhRNA | n/a | 32% | n/a | 47% |
| Tat-Env-lhRNA | n/a | 52% | n/a | 51% |
| Env-Vpu-lhRNA | n/a | n/a | 25% | 7% |
| Vpu-Env-lhRNA | n/a | n/a | 34% | 4% |
